# New Clarification About Observation Billing May Improve Care for Behavioral Health Patients

**DOI:** 10.5811/westjem.2019.11.45703

**Published:** 2020-01-27

**Authors:** Anwar D. Osborne, Matthew A. Wheatley, Christopher W. Baugh, Michael Granovsky

**Affiliations:** * Emory University, Department of Emergency Medicine, Atlanta, Georgia; † Emory University, Department of Internal Medicine, Atlanta, Georgia; ‡ Harvard University, Department of Emergency Medicine, Cambridge, Massachusetts; § Logix Health, Bedford, Massachusetts

To the Editor:

Emergency physicians (EP) provide ongoing care to psychiatric patients beyond the confines of a standard emergency department (ED) visit. Often, when we identify patients who need specialty psychiatric care, patients board in the ED awaiting acceptance and transfer to an outside facility. Even when it has taken multiple days to complete the transfer, it has been unclear how to properly obtain reimbursement for this care.

Two years ago, the American College of Emergency Physicians (ACEP) Observation Medicine Section surveyed its members as to their usual care of psychiatric patients. This small, 100 ACEP-member survey showed a byzantine distribution of care models, ranging from EPs rounding on the patients, to intermittent psychiatric re-evaluation, to no evaluations beyond medical clearance. Some 86% of respondents indicated they order medications for psychiatric patients while boarding, and a mere 46.5% of respondents use home medications in limited circumstances.

There was also significant variability in the billing for observation services related to psychiatric conditions in the ED. These services were billed by respondents almost as frequently as they were not billed (35.0% vs 31.0%), while 35.0% were unsure whether their observation services were being billed at all.

Recently, the ACEP Coding and Nomenclature Committee and the ACEP Emergency Medicine Current Procedural Terminology (CPT) representatives received clarification regarding how to report extended-stay mental health services. The ACEP Emergency Medicine CPT team submitted a typical case of a prolonged behavioral health stay to the CPT panel. CPT’s response, as described in July 2019 *CPT Assistant*,^1^–^2^ supports the use of observation coding (CPT 99218–99220 for the initial days, CPT 99224–99226 for the middle days, and 99217 for discharge day) for these patients.

There is face validity to this approach, as EDs are providing medical services and ongoing treatment to determine the need for admission during the boarding period. Just as observation services and observation units can standardize the care of patients with chest pain or transient ischemic attack, creating observation treatment pathways for boarding psychiatric patients can provide protocolled medications and re-evaluations, improving care while they await transfer. Ultimately, some patients may improve enough to be safely discharged from the ED, avoiding more costly inpatient care.

This recent clarification, while not directly reducing boarding of psychiatric patients, can improve their care, and allow EPs to get credit for their work. Bringing additional funding to a tremendously under-resourced mental health system is a step in the right direction.
